# Effects of cadmium on oxidative stress and cell apoptosis in *Drosophila melanogaster* larvae

**DOI:** 10.1038/s41598-022-08758-0

**Published:** 2022-03-19

**Authors:** Pingping Yang, Xingran Yang, Liran Sun, Xiaobing Han, Lu Xu, Wei Gu, Min Zhang

**Affiliations:** College of Life Sciences, Shaanxi Normal University, Xi’an, 710119 China

**Keywords:** Biochemistry, Cell biology, Chemical biology, Genetics, Molecular biology

## Abstract

With the increase of human activities, cadmium (Cd) pollution has become a global environmental problem affecting biological metabolism in ecosystem. Cd has a very long half-life in humans and is excreted slowly in organs, which poses a serious threat to human health. In order to better understand the toxicity effects of cadmium, third instar larvae of *Drosophila melanogaster* (Canton-S strain) were exposed to different concentrations (1.125 mg/kg, 2.25 mg/kg, 4.5 mg/kg, and 9 mg/kg) of cadmium. Trypan blue staining showed that intestinal cell damage of *Drosophila* larvae increased and the comet assay indicated significantly more DNA damage in larvae exposed to high Cd concentrations. The nitroblue tetrazolium (NBT) experiments proved that content of reactive oxygen species (ROS) increased, which indicated Cd exposure could induce oxidative stress. In addition, the expression of mitochondrial adenine nucleotide transferase coding gene (*sesB* and *Ant2*) and apoptosis related genes (*Debcl, hid, rpr, p53, Sce* and *Diap1*) changed, which may lead to increased apoptosis. These findings confirmed the toxicity effects on oxidative stress and cell apoptosis in *Drosophila* larvae after early cadmium exposure, providing insights into understanding the effects of heavy metal stress in animal development.

## Introduction

Cadmium (Cd) is not an essential element for all organisms, and its presence, even in low concentrations, can have toxic effects. In industrial settings, the inhalation or ingestion of Cd can cause acute or chronic poisoning. In humans, food is the main source of Cd exposure^1^: foods rich in Cd include grains, animal offal, seafood products, and cocoa powder^2^. Smoking is another common way of introducing Cd into the human body. Cd exposure in infants and juveniles directly affects development and growth, leading to the destruction of the testicular blood barrier, germ cell apoptosis, testicular oedema, hemorrhaging, necrosis, increased lipid peroxidation, and infertility^3^. Additionally, excessive Cd exposure can cause genomic instability, which has been linked to various cancers^4^. Thus, it is crucial to further elucidate the effects of Cd exposure, as it poses a significant risk to human health and environment.

ROS is an unavoidable product of aerobic cell activity. Aerobic activity produces numerous metabolites, under the control of a series of specific systems, and exceeding the metabolite threshold accelerates the production of ROS. High level of ROS can mediate damage to cell structures, lipids and membranes^5^, and act as signal molecules in cell differentiation, cell cycle, and cellular response to external stimuli^6^. Cd activates the mitogen-activated protein kinase (MAPK) pathway which is related to the transcription of oxidative stress signal molecules. Chen et al.^7^ and Oswald et al.^8^ studied the locomotive behavior of *Drosophila* larvae, and found that ROS acted as an obligate signal for neuronal activity in *Drosophila* motor neurons, and were structurally plastic at the presynaptic and postsynaptic ends.

When Cd-induced damage exceeds the cell’s ability to repair itself, the signal transduction pathway is activated to initiate the process of apoptosis, which can minimize damage to a local area^9^. Changes in mitochondrial membrane potential, structure, and permeability occur during apoptosis in *Drosophila*^10^. Apoptosis can maintain cellular environmental homoeostasis, and is an autonomous and spontaneous behavior that is of great significance for maintaining organism health, nervous system development, and immune system functions^11^. The Jun N-terminal kinase (JNK) is the main apoptosis-promoting factor in *Drosophila* that can activate a variety of transcription factors, such as AP-1^12^. Dying cells produce high levels of ROS and JNK,elevated JNK levels can promote cell apoptosis or increase ROS production through other mechanisms.

Cd, which enters larva through ingestion, can still be detected in *Drosophila* after complete metamorphosis and development^13^. Exposure to Cd at the larval stage of *Aedes albopictus* can reduce survival, prolong development time, and reduce the weight, reproduction, and survival rate of adults^14^. This may be due to the interaction of Cd with biological macromolecules of organism, which may disrupt the normal process of cell division, introduce oxidative stress from excessive ROS, or consume excessive energy with detoxification mechanisms.

In this study, *Drosophila melanogaster* (Canton-S strain) was used as experimental material to explore the effects of heavy metal cadmium on oxidative stress and apoptosis of third instar larvae, so as to provide a more comprehensive understanding of the toxic effects and toxicity mechanism of early cadmium exposure on *Drosophila melanogaster* larvae.

## Material and methods

### Reagents

Analytical-grade Cd chloride was purchased from Fuchen Chemical Industries Ltd. (Tianjin, China). Collagenase IV and trypan blue were purchased from Beijing Solarbio Technology Ltd. (Beijing, China). NBT, comet assay, and DNA damage detection kits were acquired from Nanjing Jiancheng Bioengineering Institute (Nanjing, China). SYBR Green I and premix Taq DNA polymerase were obtained from Hunan Aikerui Biological Engineering Ltd. (Changsha, China). The RNAiso Plus Kit was acquired from Beijing Baori Medical Biotechnology Ltd. (Beijing, China).

### Determination of LC_50_ of Cd to ***Drosophila*** larvae

Using the maximum Cd concentration permitted by the *National Standard for Food Safety* (GB-2762-2017) as a reference point, the median lethal concentration (LC_50_) was determined by different culture mediums with nine Cd concentrations: 0 mg/kg (control), 1 mg/kg, 2 mg/kg, 4 mg/kg, 8 mg/kg, 16 mg/kg, 32 mg/kg, 64 mg/kg, and 128 mg/kg.

Male and female *Drosophila* were collected within 8 h after eclosion and were cultured separately for three days^15^. They were then placed together in activated carbon culture media, and allowed to mate for 12 h. The eggs were picked and cultured in culture mediums with different cadmium concentrations. There were 175 eggs per concentration group. Eclosion rate which is the proportion of eclosed adults from larvae was calculated. The experiments were performed in triplicate and the results were averaged.

### Toxicity determination

Based on the results of LC_50_ experiment, four cadmium concentrations (1.125 mg/kg, 2.25 mg/kg, 4.5 mg/kg, and 9 mg/kg) were set up for subsequent experiments. Similar to the LC_50_ experiment, male and female *Drosophila* were collected within 8 h after eclosion and cultured separately for three days. They were then crossed in fresh control medium (without Cd) or Cd mediums for 12 h. Parental adult flies were then removed, and third instar larvae developed from different medium were selected for the experiments.

NBT reduction test was used to detect the ROS content in *Drosophila* larvae cells^16^. Larvae haemolymph cells (extracted from fifty larvae per sample) were incubated with NBT for 1 h in the incubation medium. The insoluble formazan crystals formed were solubilized by adding 150 µL 50% acetic acid followed by 5 min vortexing. The absorbance of the final solution was measured at 595 nm using a spectrophotometer.

Trypan blue staining was used to detect cellular damage in the midgut of larvae^17^. Seventy third instar larvae were randomly selected from each treatment group, cleaned with PBS, put in 0.02% trypan blue staining solution, and incubated for 2 h with vibration. After cleaned with PBS, the samples were checked and photographed under a stereomicroscope.

Crawling experiment was used to identify the neuronal damage in the early stage of larval development. Three instar larvae were randomly selected from Cd medium with different concentrations and washed in PBS solution, then placed in the center of Petri dish containing 0.8% agarose gel. A layer of grid paper was put under the Petri dish. After the larvae adapted for 10 min, the crawling track, turning angle and number of peristalsis contractions (full anterior to posterior movement = 1 contraction) of larvae within 2 min were recorded^18,19^.

Comet assay can effectively detect and analyze the degree of DNA damage in cells^20^. Fifty third instar larvae were randomly selected from each group. The midgut was dissected under an anatomical microscope, digested in collagenase solution for fifteen minutes, and the cells were collected by centrifugation, washed with PBS and centrifuged to make cell suspension. Then the experiment was performed according to the instructions of the DNA Damage Detection Kit (Jiangsu KeyGEN BioTECH Corp., Ltd, China). Finally, the samples were checked under the fluorescence microscope (Carl Zeiss Jena CO., LTD, Germany) (515–560 nm wavelength).

### Quantitative real-time PCR

Total RNA from six third instar larvae was isolated using the RNAiso Plus Kit (TAKARA, Beijing, China), according to the manu-facturer’s instructions. The coding sequence (CDS) sequences of two mitochondrial adenine nucleotide transferase (ANT) coding genes (*sesB* and *Ant2*) and six apoptosis-related genes (*Debcl*, *hid*, *rpr*, *p53*, *Sce*, and *Diap1*) were queried using NCBI (https://www.ncbi.nlm.nih.gov/). Primer design and synthesis were carried out at the Shenggong Bioengineering Co. Ltd., Shanghai. cDNA was obtained via RNA reverse transcription, following the instructions of the PrimeScript ®RT Master Mix reverse transcription kit. The reaction system was prepared according to the instructions of the SYBR Green kit to detect the mRNA expression of the aforementioned genes.

The qPCR protocol used was as follows: following initial denaturation at 95 ℃ for 30 s, the template cDNA was amplified for 40 cycles of denaturation (95 ℃, 5 s), annealing (60 ℃, 34 s), and extension (75 ℃, 45 s). The experiments were performed in triplicate and the results were averaged. The amplification primers are shown in Table 1.

**Table 1:** Primers for real-time quantitative PCR amplification.

Gene	Sense primer 5′–3′	Anti-sense primer 5′–3′
*Rp49*	GCTAAGCTGTCGCACAAA	TCCGGTGGGCAGCATGTG
*sesB*	CTCGCTTGGCTGCTGATACTGG	GGCGGCACGGTAGATGATGATG
*Ant2*	GTGGGCAAGGGCGGCAATC	AACGGTGTGCTCTTCGGATTCG
*Debcl*	ATCCGCTCCGCTCCGAGTG	CCCGCTATTGTTGCTGCTGAGG
*hid*	ACTGGCGGCGATGTGTTCTTTC	TTGGCTGCGGTGTTGATGGC
*rpr*	TCGCAAGCCATCGCAATGAGG	CGGCTGATGAGTGGTGACTGTG
*p53*	AGCCGAGACTGCGACGACTC	GCCGCCGCCTCCTTAATCATG
*Sce*	GCCATGTCGGTGCTGACTTCG	TTGCCCTCGCCCTCTGTGTC
*Diap1*	GTTGCGGCGGTGGTCTCATG	AGCTTGACGAATCGGCACTGAC

### Statistical analysis

All the experimental data were analyzed by SPSS 20.0 statistical software (IBM Company, New York, America) using one-way ANOVA and Tukey’s significance testing (*P* < 0.05). GraphPad Prism 7.0 (GraphPad Software Company, California, America) was used to create all graphs.

The images obtained from the comet assay were analyzed by Comet Assay Software Project (Beijing Biolaunching Technologies Co., Ltd., Beijing, China), and the tail length (TL, µm), tail moment (TM), olive tail moment (OTM), and tail DNA % (TD) were determined.

### Ethics declarations

All applicable international, national, and/or institutional guidelines for the care and use of animals were followed.

## Results

### LC_50_ value of Cd to ***Drosophila*** larvae

**Figure 1:**
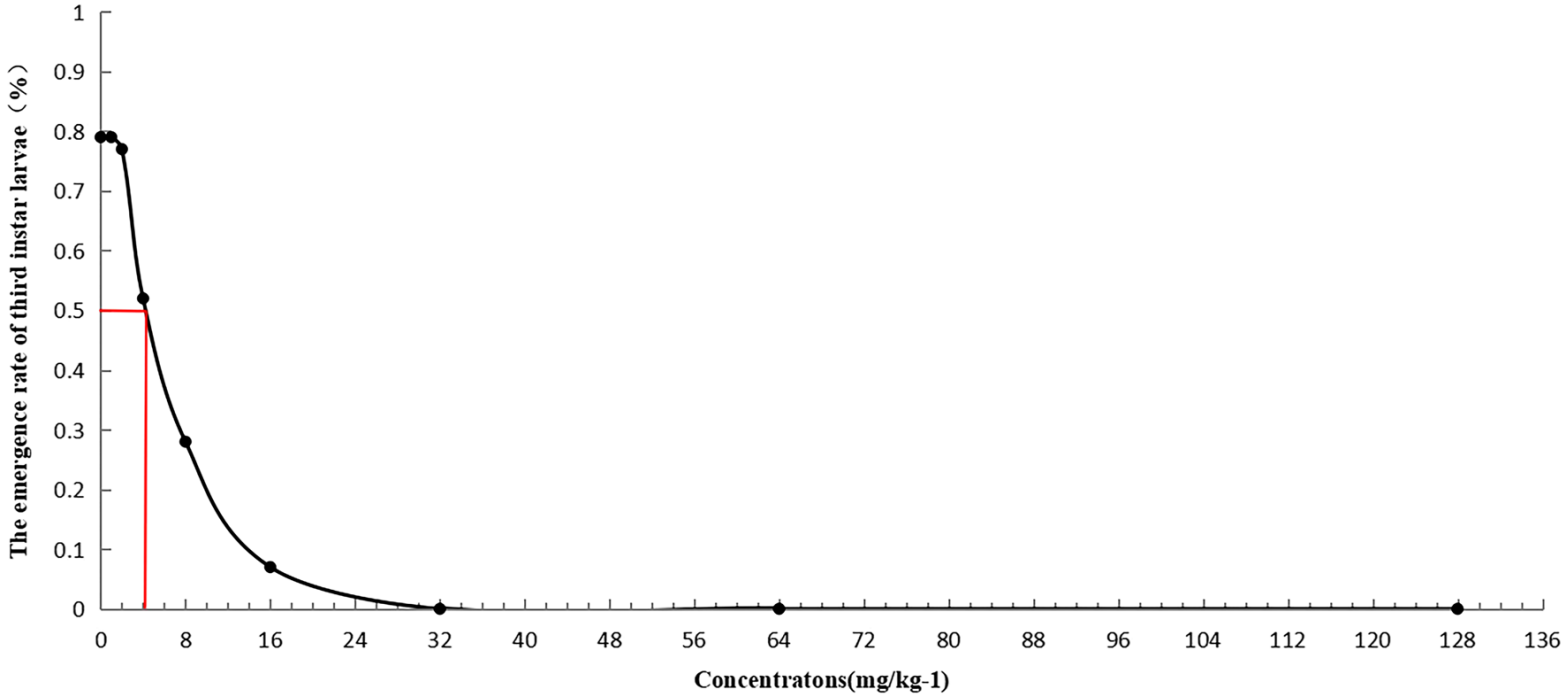
The survival curve and median lethal concentration (LC_50_) of *Drosophila* larvae. *Drosophila* larvae were exposed to a range of Cd concentrations (Mean ± SD, n = 3, 175 eggs per sample). Cd concentration corresponding to 50% mortality was 4.449 mg/kg at the 95% confidence limit.

The eclosion rate of *Drosophila* from larvae under different Cd concentrations was presented in Fig. 1. These results showed that the eclosion rate decreased with increasing Cd concentration, that is, the mortality increased gradually. The Cd concentration corresponding to 50% mortality was 4.449 mg/kg at the 95% confidence limit. LC_50_ was used as the basis for setting the cadmium concentration in the follow-up study.

### Toxicity effects of Cd on *Drosophila* larvae

#### ROS content

**Figure 2:**
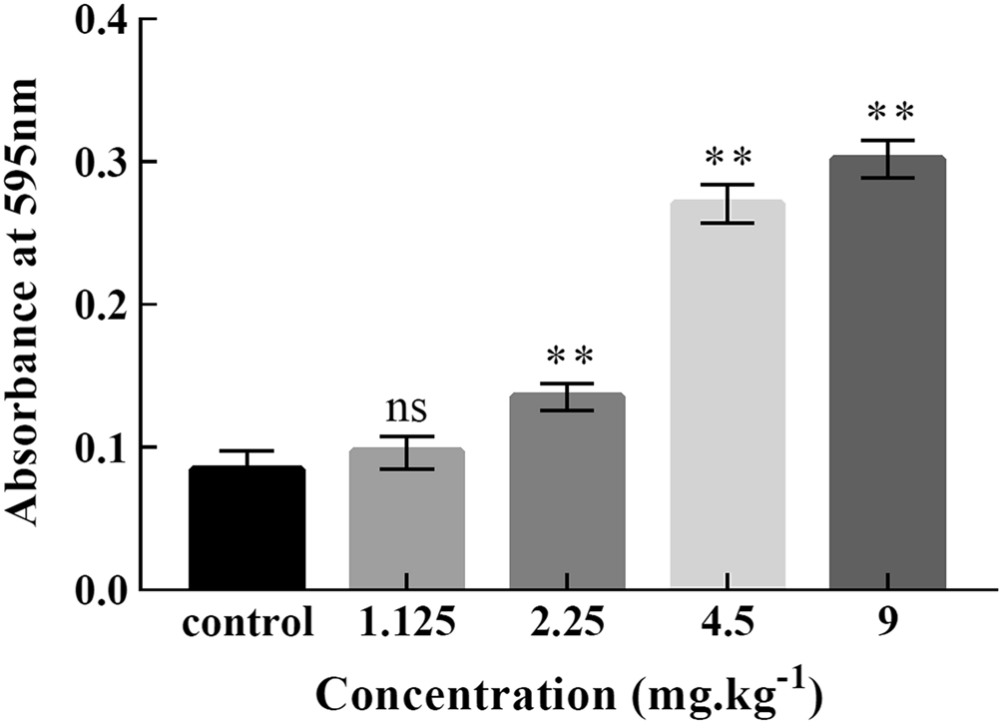
ROS in *Drosophila* larvae increased under higher concentration of cadmium stress. Nitroblue tetrazolium reduction assay for the estimation of reactive oxygen species (ROS) in *Drosophila* larvae exposed to Cd (Mean ± SD, n = 3, 50 larvae per sample, **P < 0.01, ns = not significant). The absorbance values of treatment groups of 2.25 mg/kg, 4.5 mg/kg and 9 mg/kg have extremely significant differences compared with the control group (*P* < 0.01).

The ROS content of blood lymphocytes of third instar larvae was shown in Fig. 2. Compared with the control group, the low Cd concentration group of 1.125 mg/kg has no significant difference. The absorbance values of treatment groups of 2.25 mg/kg, 4.5 mg/kg and 9 mg/kg show significant differences compared with the control group (*P* < 0.01), and the ROS level increased gradually with the increase of Cd concentration. These results showed that higher concentration Cd stress could induce a significant increase in ROS content in *Drosophila* larvae. These results showed that the content of ROS in *Drosophila* larvae increased significantly under higher concentration of cadmium stress.

#### Midgut cell damage

**Figure 3:**
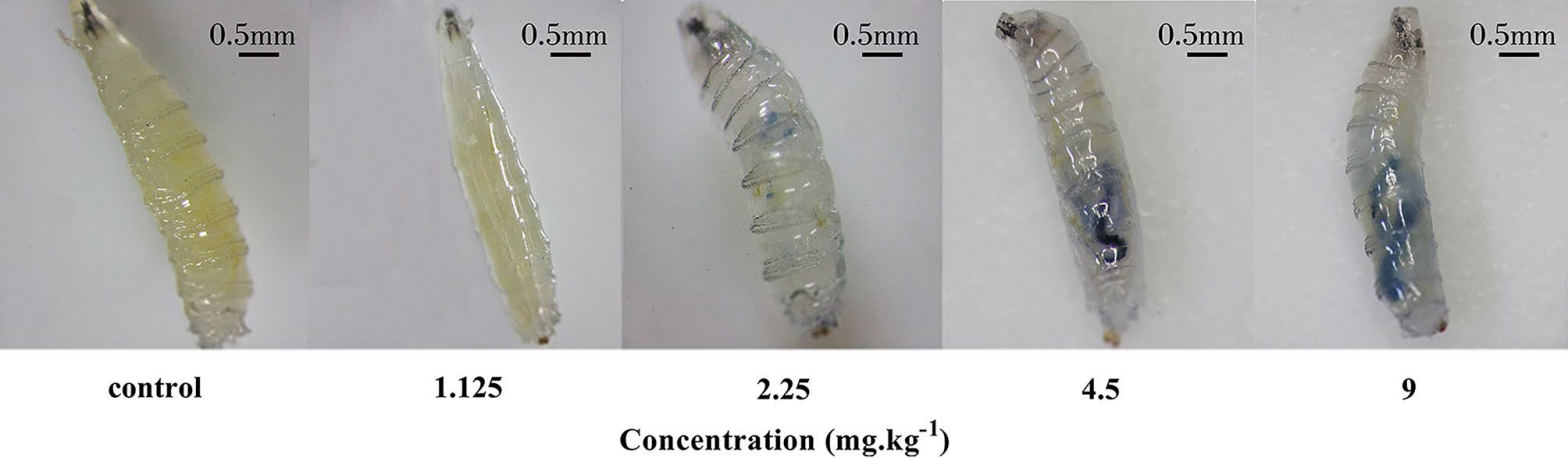
Cell damage in *Drosophila* larvae under the cadmium exposure. Representive photos of *Drosophila* third instar larvae with trypan blue staining. Trypan blue staining signal level indicated the damage degree of midgut cells.

The third instar larvae after trypan blue staining were showed in Fig. 3. Midguts of third instar larvae picked randomly in each treatment group appeared stained of varying degrees. The larger the staining area, the darker the color, indicated that the more serious the cell damage. Moreover, the results also showed that the proportion of stained *Drosophila* larvae was higher with increasing concentrations of cadmium (data not shown). These results indicated that the midgut cells of *Drosophila* larvae showed cell damage under the cadmium exposure, and the degree of damage increased dose-dependently.

#### Crawling behavior

**Figure 4:**
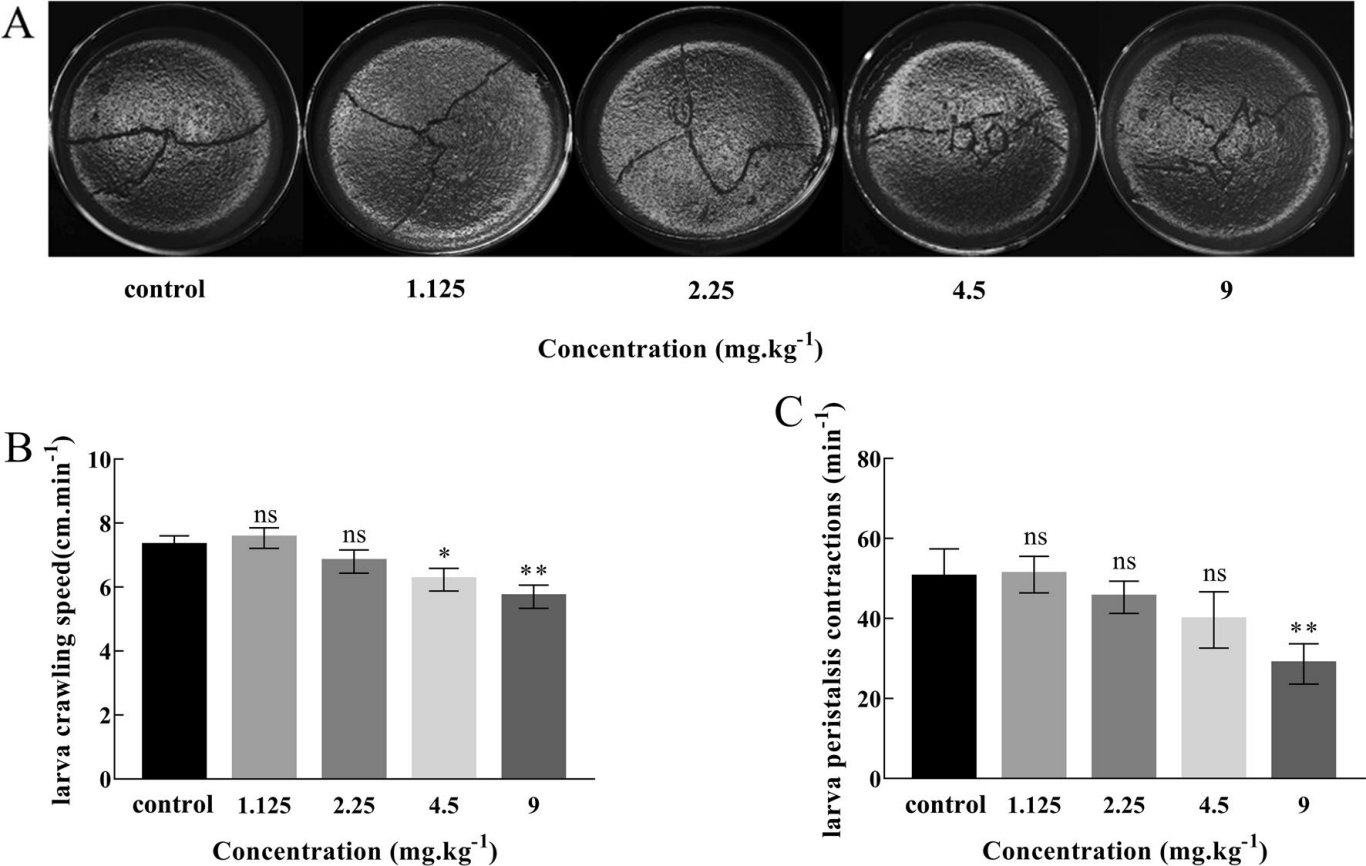
Crawling behavior of third instar larvae of *Drosophila*. (**A**) Photographs of third instar larvae crawling route. (**B**) Larvae crawling speed at increasing Cd concentrations. (**C**) Larvae peristalsis contractions at increasing Cd concentrations. (Mean ± SD, n = 3, 15 larvae per sample, *P < 0.05, **P < 0.01, ns = not significant).

As shown in Fig. 4, the crawling trajectory of larvae in the control group was smooth and simple. As Cd concentration increased, the larvae crawling trajectory became increasingly irregular, with many sharp or circular turns (Fig. 4A). Crawling speeds of larvae in the 4.5 mg/kg (*P* < 0.05) and 9 mg/kg (*P* < 0.01) treatment groups were significantly lower than that of the control group (Fig. 4B). Additionally, larvae exposed to 9 mg/kg had a significantly lower number of body wall contractions compared with the control group (*P* < 0.01) (Fig. 4C). These results showed that high concentrations of cadmium induced abnormal behavior of *Drosophila* larvae, including abnormal turns and twists, crawling speed reduction and abnormal body wall contraction.

#### DNA damage

**Figure 5:**
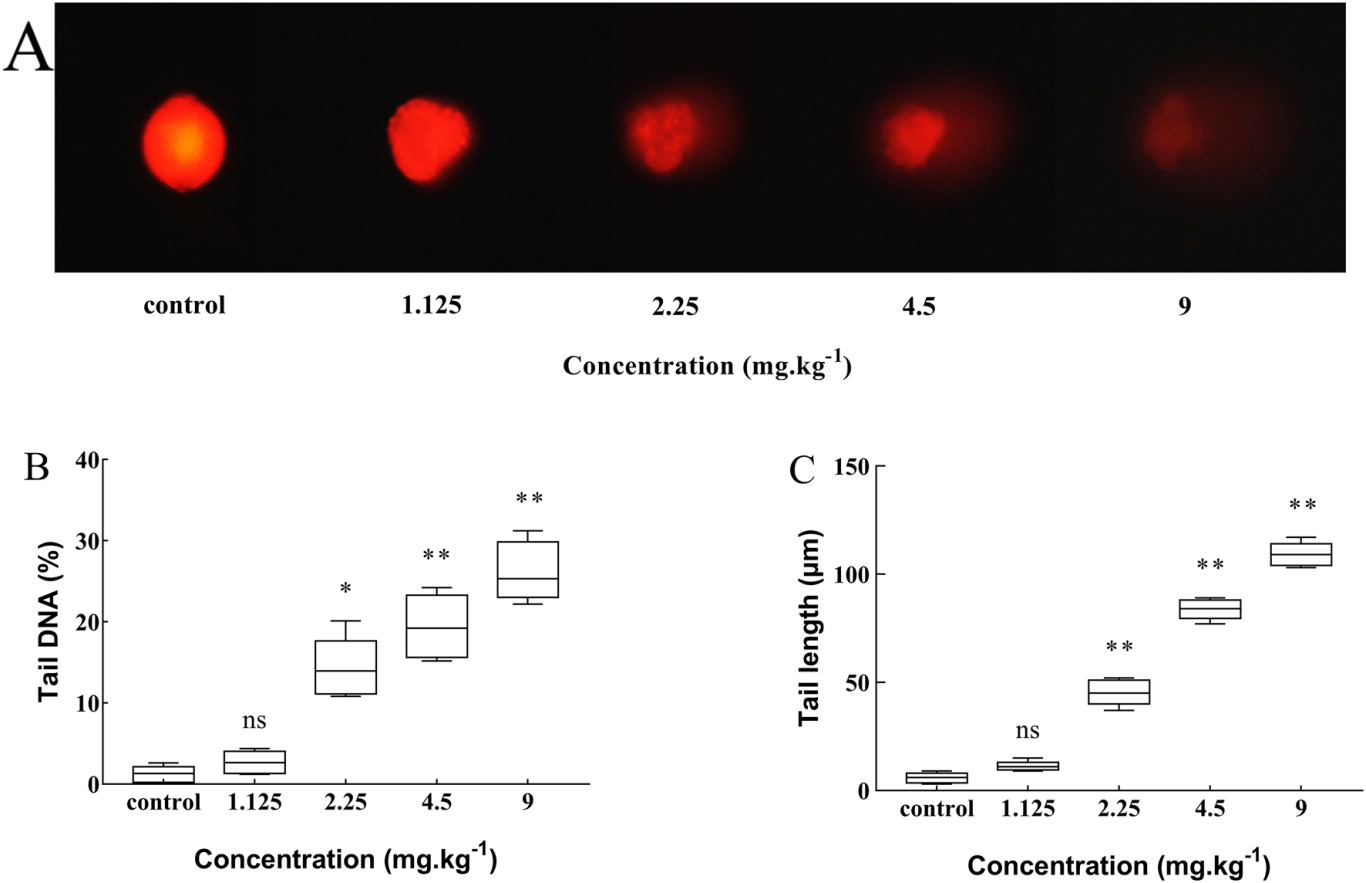
DNA damage in the midgut cells of *Drosophila* third instar larvae under Cd stress. (**A**) Comet images of midgut cells in third instar larvae indicated the DNA “smearing” phenomenon of *Drosophila* larvae was serious with the increase of Cd concentration. (**B**) Tail DNA% of comet at increasing Cd concentration. (**C**) Tail length of comet at increasing Cd concentration. The increase of tail DNA% and tail length of comet indicating clear DNA damage in treatment groups (2.25 mg/kg, 4.5 mg/kg, 9 mg/kg). (Mean ± SD, n = 3, 50 larvae per sample, *P < 0.05, **P < 0.01, ns = not significant).

The images of comet assay of midgut cells of the third instar larvae were shown in Fig. 5A. Tail DNA% of comet and tail length of comet were shown in Fig. 5B, C respectively. Tail DNA% and tail length are used to assess the degree of DNA damage in comet assay. According to the grade of DNA damage, tail DNA% in the control group was not damaged. Treatment groups of 2.25 mg/kg and 4.5 mg/kg belonged to mild damage, group of 9 mg/kg was moderately damaged. Tail DNA% of comet was significantly increased in the three treatment groups compared with the control group (*P* < 0.05 or *P* < 0.01). The tail length of comet of the treatment groups (2.25 mg/kg, 4.5 mg/kg and 9 mg/kg) increased significantly compared with the control group (*P* < 0.01). These results indicated that the DNA “smearing” phenomenon became more severe, and there was a higher degree of DNA damage in *Drosophila* larvae with the increase of cadmium concentration.

### Effects of Cd on gene expression of *Drosophila* larvae

**Figure 6:**
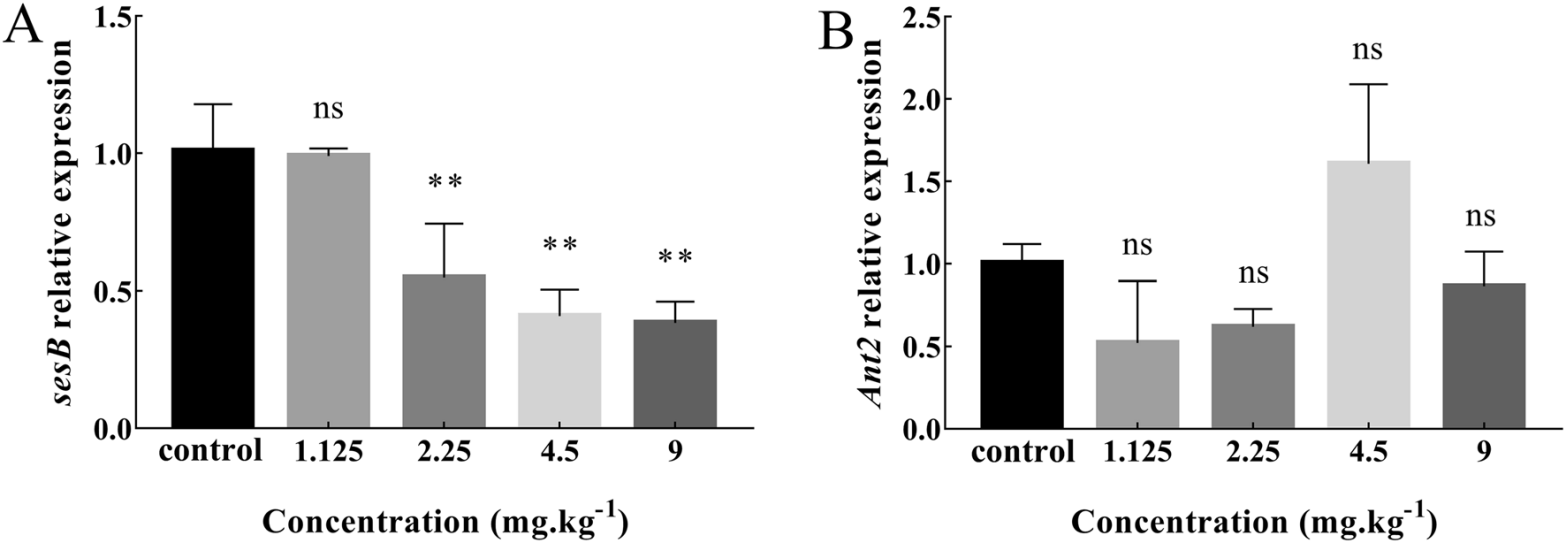
Effects of Cd on the gene expression of adenine nucleotide transferase coding genes *sesB* (**A**) and *Ant2* (**B**) in *Drosophila* third instar larvae (Mean ± SD, n = 3, 6 larvae per sample, **P < 0.01, ns = not significant). The mRNA expression of *sesB* in treatment groups reduced significantly compared with the control group (*P* < 0.01). There was no significant difference in mRNA expression level of *Ant2* of Cd treatment groups compared with the control group (*P* > 0.05).

The increase of ROS in *Drosophila* is closely related to mitochondria. ANT protein is a transporter related to apoptosis in mitochondrial transport family. ANT is jointly encoded by *Ant1* (*sesB*) and *Ant2* in *Drosophila*^21^. Effects of Cd on the gene expression of adenine nucleotide transferase coding genes *sesB* and *Ant2* in *Drosophila* larvae were shown in Fig. 6. The expression of *sesB* in treatment groups of 2.25 mg/kg, 4.5 mg/kg and 9 mg/kg decreased significantly compared with the control group (*P* < 0.01). The expression level of *Ant2* appeared to decline first, then increase and then decrease, but there was no significant difference compared with the control group (*P* > 0.05). These results showed that Cd exposure inhibited the expression of *sesB* gene in *Drosophila* larvae.

**Figure 7:**
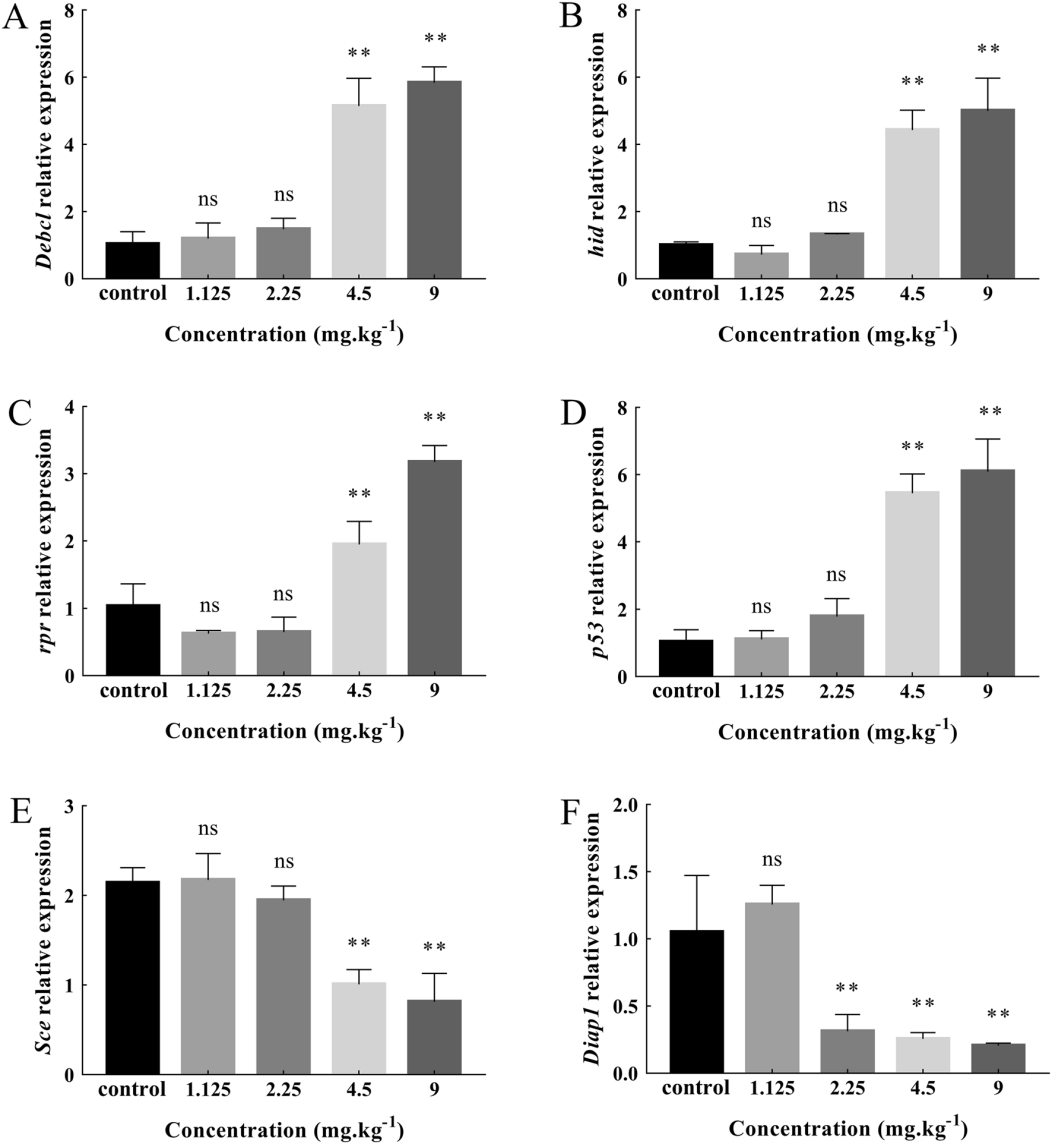
Effects of Cd on the gene expression of pro-apoptotic and anti-apoptotic genes in *Drosophila* third instar larvae. Real time PCR results showing the expression of pro-apoptotic genes, (**A**): *Debcl*; (**B**): *hid*; (**C**): *rpr*; (**D**): *p53*; (**E**): *Sce*; (**F**): *Diap1*; (Mean ± SD, n = 3, 6 larvae per sample, **P < 0.01, ns = not significant). Compared with the control the mRNA expression of *Debcl*, *hid*, *rpr*, and *p53* increased at higher Cd concentrations (4.5 mg/kg and 9 mg/kg) (*P* < 0.01) (**A–D**). The expression of the anti-apoptotic gene *Sce* was significantly reduced at higher Cd concentrations (4.5 mg/kg and 9 mg/kg) (*P* < 0.01) (**E**) . The expression of anti-apoptotic gene *Diap1* showed a downward trend and decreased significantly in 2.25 mg/kg, 4.5 mg/kg and 9 mg/kg treatment groups compared with the control group (*P* < 0.01) (**F**).

Increased ROS induced by Cd stress will lead to oxidative stress and apoptosis. Therefore, we also detected the expression changes of pro-apoptotic genes (*Debcl*, *hid*, *rpr*, *p53*) and anti-apoptotic genes (*Sce*, *Diap1*) in *Drosophila*. As shown in Fig. 7, the expression of pro-apoptotic and anti-apoptotic genes changed in third instar larvae under different Cd concentrations. The expression of pro-apoptotic genes, *Debcl*, *hid*, *rpr*, and *p53* (Fig. 7A–D), increased with increasing Cd concentrations. Compared with the control group, the expression of pro-apoptotic genes in the two highest concentration treatment groups (4.5 mg/kg and 9 mg/kg) increased significantly (*P* < 0.01). The expression of the anti-apoptotic gene *Sce* was significantly downregulated in the 4.5 mg/kg and 9 mg/kg treatment groups (*P* < 0.01) (Fig. 7E). The expression of *Diap1* showed a downward trend and decreased significantly in 2.25 mg/kg, 4.5 mg/kg and 9 mg/kg treatment groups compared with the control group (*P* < 0.01) (Fig. 7F).

## Discussion

In this study, trypan blue staining showed that *Drosophila* intestinal cell damage increased with increasing exposure to Cd. The NBT experiments proved that ROS increased after Cd treatment. As excessive ROS has been shown to induce DNA damage^22^, it is unsurprising that the comet assay analysis indicated significantly more DNA damage in *Drosophila* exposed to high Cd concentrations. Cd could interact with ROS regulators, such as glutathione, reducing the ability to eliminate ROS, indirectly increasing ROS levels and inducing oxidative stress^23^. Previous studies have found that heavy metal exposure caused a decrease in catalase (CAT) and superoxide dismutase (SOD) activity and an increase in MDA content in *Drosophila* larvae^24^. This leads to the accumulation of ROS in cells, which can cause oxidative stress and subsequent DNA damage.

Our results indicated that larvae in the high Cd concentration groups exhibited abnormal crawling behavior compared with the control group. Similarly, other studies have found that *Drosophila* eggs treated with 250 and 300 mg graphene/L resulted in a significantly reduced crawling speed^25^, and those treated with high concentrations of NaF showed significantly reduced crawling speed and body wall contractions^19^. The abnormal crawling behavior of larvae exposed to high Cd concentrations indicates that mechanical motor neurons are affected by toxic stress, which may be caused by brain coordination defects and damage to the subesophageal ganglion. The slower crawling speed may be caused by damage to the cholinergic neurons, which can result in abnormal muscle contraction.

This study also identified that the relative expression of *sesB* in the third instar larvae of *Drosophila* decreased with increasing Cd concentration. *sesB* primarily encodes the mitochondrial ANT protein: mitochondria are the only source of adenylate triphosphate (ATP) within the synapses, therefore, ANT is essential for the transport of ATP from the mitochondrial membrane to the cytoplasmic matrix^26^. DeVorkin et al.^27^ found that *Drosophila* caspase1 (*Dcp-1*) is located upstream of *sesB,* and can promote autophagy in the salivary glands, midgut, and other parts of larvae by inhibiting *sesB* activity. Thus, *sesB* was shown to be a new negative regulator of autophagic flux in the mid-stage of *Drosophila* development. With a decrease in *sesB* expression, the mitochondrial morphology changed from elongated organelles to spherical particles, causing changes in their metabolic state and possibly inducing cell apoptosis^28^. The remaining mitochondria maintain tissue viability through increased activity, which can lead to an increase in ROS. When *sesB* was inhibited, the H_2_O_2_ content increased sharply, and O_2_− was produced by the G3PDH active enzyme and ubiquinone on the cytoplasmic side of the inner mitochondrial membrane^29^. This indicates that O_2_− /H_2_O_2_ are created by a phosphoglyceride shuttle in the body of *Drosophila*, making *sesB*-knockout cells more sensitive to oxidative stress. *sesB* also regulates calcium levels within cells and mitochondria; therefore, when *sesB* was knocked out, calcium signaling in mitochondria was blocked^30^. Changes in *sesB* expression generally impair mitochondrial function, leading to reduce toxic metabolism, oxidative damage, and even cell death^31^.

*Debcl* has been shown to affect a wide range of other genes and functions. *Debcl* inhibits the expression of Buffy^32^, a gene involved in stress- and DNA damage-induced apoptosis that is not required for normal development in *Drosophila*. Additionally, the tumor suppressor gene, *Rbf1*, interacts with *Debcl* to increase the amount of ROS produced by mitochondria, leading to the activation of the JNK pathway and, eventually, to apoptosis in *Drosophila*^33^. *Debcl* may also increase mitochondrial ROS content through glycerophosphate oxidase 1^34^. Overexpression of *Debcl* in the eyes of *Drosophila* also leads to eye-cell apoptosis^35^. Based on these previous findings and the results of this study, it can be presumed that the upregulation of *Debcl* expression may ultimately induce apoptosis by increasing the activity of glycerophosphate oxidase 1^34^ and the mitochondrial fission protein Drp1^36^. ROS content may be further increased by the increase in Gpo-1 expression, caused by the upregulation of *Debcl* genes in the Bcl-2 family.

*p53* can promote DNA repair; however, when DNA damage repair is incomplete or fails, it can promote apoptosis instead^37^. The upregulation of *p53* may additionally stimulate the upregulation of other pro-apoptotic genes, *rpr* and *hid* in the RHG family, and inhibit the expression of the anti-apoptotic gene, *Diap1*^38^. Accordingly, in addition to the upregulation of *p53*, the expression of *rpr* and *hid* were also upregulated after Cd stress in this study, whereas the *Diap1* expression was significantly downregulated. *Diap1* is a key enzyme that controls cell fate, and regulates apoptosis by selectively degrading pro-apoptotic proteins or itself^36^, helping to prevent apoptosis caused by abnormal caspase activation. However, the protein produced by *rpr* and *hid* inhibits the expression of *Diap1* and induces the release of Dronc (a caspase-9 homolog). After Dronc is released, it binds to the scaffold protein Ark (Apaf-1) and hydrolysis activates Drice, *Dcp-1* (a caspase-3 homolog)^37^. These caspases induce cell DNA fragmentation and the formation of apoptotic bodies^39^. This study indicated that Cd stress induced the upregulation of pro-apoptotic genes (*Debcl*, *hid*, *rpr,* and *p53*) and the downregulation of anti-apoptotic genes (*Sce* and *Diap1*) at the mRNA level in *Drosophila* larvae. Changes in the expression of these genes may also change the membrane potential in the mitochondria and lead to a harmful increase in ROS content and further DNA damage.

## Conclusions

Heavy metal cadmium induced damage of midgut cells of third instar larvae in *Drosophila melanogaster*. The DNA ‘smearing’ phenomenon indicated DNA damage induced by cadmium exposure. Moreover, the content of reactive oxygen species increased, suggesting oxidative stress. Cadmium could also lead to behavioral changes of Drosophila larvae, such as excessive distortion, increased turning times and slower crawling speed. In addition, Cd induced changes in the expression of adenine nucleotide transferase coding gene *sesB* in *Drosophila* larvae, suggesting that Cd stress may produce more ROS by affecting the functional changes of mitochondria. The relative expression of pro-apoptotic genes *Debcl*, *hid*, *rpr* and *p53* increased, meanwhile, anti-apoptotic genes *Sce* and *Diap1* decreased. In conclusion, cadmium exposure may induce oxidative stress and apoptosis in *Drosophila* larvae by increasing the content of reactive oxygen species. Consistent with previous studies^40,41^, this study provided further evidence about the toxicity effects of early cadmium exposure on *Drosophila* larvae. The present study suggests that cadmium exposure induces oxidative stress and cell apoptosis, which could be one of the main mechanisms for induction of genotoxicity.

## Data Availability

All data included in this study are available upon request by contact with the corresponding author (zhangmin451@snnu.edu.cn).
